# UAV Swarm Navigation Using Dynamic Adaptive Kalman Filter and Network Navigation

**DOI:** 10.3390/s21165374

**Published:** 2021-08-09

**Authors:** Jingjuan Zhang, Wenxiang Zhou, Xueyun Wang

**Affiliations:** School of Instrument Science and Optoelectronics Engineering, Beihang University, XueYuan Road No. 37, HaiDian District, Beijing 100191, China; zhangjingjuan@buaa.edu.cn (J.Z.); 17806286806@buaa.edu.cn (W.Z.)

**Keywords:** adaptive Kalman filter, ensemble empirical mode decomposition, process noise, observation noise, networked navigation

## Abstract

Aiming to improve the positioning accuracy of an unmanned aerial vehicle (UAV) swarm under different scenarios, a two-case navigation scheme is proposed and simulated. First, when the Global Navigation Satellite System (GNSS) is available, the inertial navigation system (INS)/GNSS-integrated system based on the Kalman Filter (KF) plays a key role for each UAV in accurate navigation. Considering that Kalman filter’s process noise covariance matrix Q and observation noise covariance matrix R affect the navigation accuracy, this paper proposes a dynamic adaptive Kalman filter (DAKF) which introduces ensemble empirical mode decomposition (EEMD) to determine R and adjust Q adaptively, avoiding the degradation and divergence caused by an unknown or inaccurate noise model. Second, a network navigation algorithm (NNA) is employed when GNSS outages happen and the INS/GNSS-integrated system is not available. Distance information among all UAVs in the swarm is adopted to compensate the INS position errors. Finally, simulations are conducted to validate the effectiveness of the proposed method, results showing that DAKF improves the positioning accuracy of a single UAV by 30–50%, and NNA increases the positioning accuracy of a swarm by 93%.

## 1. Introduction

In recent years, the unmanned aerial vehicle (UAV) swarm has been given more and more attention due to the tremendous application prospects. Compared with a single UAV, the UAV swarm is more efficient, reliable, flexible and intensive [1,2]. As a prerequisite for a UAV to complete various tasks, the navigation and positioning technology has been extensively studied by scholars. At present, the most popular navigation system usually consists of an inertial navigation system (INS) and a Global Navigation Satellite System (GNSS) [3]. This system is very effective for improving navigation accuracy; however, its disadvantage of a fragile robustness due to the easy loss or disturbance of the GNSS signal limits its applications. When the GNSS signal is available, the Kalman filter (KF) is the most popular INS/GNSS data fusion algorithm. This paper aims to improve the fusion accuracy of the traditional KF and solve the problem of the UAV swarm navigation in the case of GNSS outage.

The Kalman filter uses the next observation to update the estimates of the current state variables, along with their uncertainties. KF corrects the noisy sensor data to a more realistic sensor output (with less noise) [4]. However, the performance of KF is affected by the stochastic model describing the process noise and observation noise and the dynamic model describing the dynamics of the system over time [5]. A variety of adaptive Kalman filters (AKF) has been proposed by scholars to improve the fusion precision of traditional Kalman filters when the stochastic model is inaccurate [6]. The AKF based on the attenuation factor reduces the influence of historical data on the current results by adjusting the weight of the one-step prediction covariance matrix. In fact, the problem of the inaccurate noise model has not been solved [7]. In most adaptive Kalman filtering algorithms, one of Q and R is usually fixed to adjust the other adaptively. For example, Li et al. proposed an adaptive Kalman filter based on the ensemble empirical mode (EMD) for X-ray pulsar navigation on the premise that the model has an accurate process noise covariance matrix Q [8]. The adaptive Kalman filter algorithm based on an innovation sequence and pseudo-measurement vector method proposed by Zhang [9] and the improving adaptive Kalman estimation algorithm based on a covariance-matching technology mentioned in [10] are implemented on the assumption that R is completely known. The multi-model adaptive Kalman filter can estimate the unknown probability of measurement loss, but its premise is that both Q and R are clear and the calculation of the algorithm requires multiple Kalman filters [11]. However, these three premises are difficult to achieve in practical applications. 

Unfortunately, in case of GNSS outage, the INS/GNSS-integrated system does not work. The UAV position estimation based solely on the INS will drift significantly over time [12]. At present, the artificial neural network (ANN) is the mainstream solution to deal with this situation. The introduction of the ANN was essentially to establish a nonlinear relationship between different inputs and outputs, and then estimate and correct inertial navigation errors when the GNSS is unavailable [13]. For example, the author uses the position information output by the INS and the output of the interactive multi-model extended Kalman filter (IMM-EKF) to train the extreme learning machine when the GNSS is available. This training model can compensate for divergent position errors during GNSS outage [14]. Wei et al. trained a wavelet neural network based on a random forest regression to provide the observation information for the AKF during a GNSS outage [15]. Chen et al. introduced a wavelet neural network (WNN) to establish the time-varying characteristic model of INS error [16]. In this system, historical error data were used to predict the current error when the GNSS signal was blocked. Abdolkarimi et al. [17] introduced a neural network based on an extreme learning machine to compensate for INS errors during a GNSS outage, and improve the signal-to-noise ratio of the sensor measurements through wavelet transform. An ensemble learning method based on the multilayer perceptron (MLP) is proposed to provide a continuous position estimation for ships when GNSS signal is unavailable [18]. However, as we all know, the prediction result of the neural network depends heavily on its own structure and training data. In addition, a lot of training time is also necessary to maintain a satisfactory accuracy.

The purpose of this paper is to provide a navigation solution that meets multiple situations (with or without GNSS). The main contents are as follows:

First, aiming at the situation that R and Q will affect each other when the adaptive Kalman filter works [10], the dynamic adaptive Kalman filter (DAKF) based on ensemble empirical mode decomposition (EEMD) is designed. This method applies EEMD to the noise extraction of multi-channel GNSS signals and estimates the noise variance to dynamically adjust the observation noise covariance matrix R. Then, through the comparison of the actual and theoretical innovation sequences, Q is adaptively modified [10]. Through the above work, the influence of the uncertain stochastic model on the performance of KF will be eliminated. A simulation will show that the DAKF has an effective improvement compared with the traditional KF.

Second, a network navigation algorithm (NNA) based on the distances among all UAVs is used. If a GNSS outage occur, the UAV swarm will automatically adjust from integrated navigation to network navigation. The NNA applies the translation–rotation approach between the measurement network with measured distances between nodes as edges and the inertial position network with inertial distances calculated from an INS output as edges to estimate the inertial positioning errors of each node in the swarm [19]. The simulation will verify that NNA can significantly improve the UAV swarm positioning accuracy.

The main contributions of this paper are as follows:

Compared with the adaptive Kalman filter algorithm mentioned in [7,8,9,10,11], the algorithm proposed in this paper can simultaneously estimate Q and R. This estimate changes dynamically with time and has a strong robustness to changing noise. The proposed algorithm also guarantees the positive semi-definiteness of the estimated state covariance matrix by using its Joseph’s form. In addition, compared with the EMD-based adaptive filtering method mentioned in [8], the proposed algorithm has the following advantages: (1) EMD suffers from the problem of mode mixing, which causes insufficient decomposition of the original signal. The EEMD algorithm introduced in this paper is not bothered by this problem [20]. This paper may be the first approach to introduce EEMD into the parameter solving of the Kalman filter in the field of integrated navigation. (2) All historical data were used for noise extraction in [8], which will lead to that R cannot effectively follow the changes of environmental noise. The proposed algorithm adds a sliding window for noise extraction so that R can better respond to the changes of real noise. (3) The noise is calculated by the fixed number of the intrinsic mode function (IMF) components in [8], which is unrealistic. The proposed algorithm uses the power spectral density (PSD) feature of the IMF to select different components to reconstruct the noise signal in different environments, which is adaptive [21]. Compared with the multi-model adaptive Kalman filter in [11], the algorithm proposed in this paper only needs one Kalman filter, which will save resources and time for calculation.

Compared with the neural network-based algorithm mentioned in [14,15,16,17,18], this paper introduces the NNA to compensate the divergent position error of the UAV swarm during a GNSS outage. The proposed algorithm does not require a large number of network parameters and training resources, and the compensation result is not affected by historical data. In addition, this paper designs a simulation to prove the superiority of the proposed algorithm compared with the neural network-based algorithm.

The rest of the paper is organized as follows. Section 2 introduces the EEMD and the adaptive Kalman filter. Then, a dynamic adaptive Kalman filter algorithm is derived. Section 3 describes the principles of the networked navigation algorithm. Section 4 provides the test results and analysis to show the superiority of the proposed methods. The conclusion is given in Section 5.

## 2. Dynamic Adaptive Kalman Filter

When the observation noise covariance matrix matches the real noise, the adjustment of the process noise covariance matrix can help the KF perform better. However, in practical applications, it is extremely difficult to obtain the real noise which will vary with the measurement environment and sensor characteristics in the measurement process [22]. This situation not only damages the performance of the Kalman filter itself, but also leads to an erroneous adjustment of Q, which makes the performance of the AKF inferior to the ordinary KF. 

In order to solve this problem, a new adaptive algorithm that can simultaneously adjust Q and R in real time is introduced. When the GNSS signal changes under the environment influence or intentional jamming, the observation noise possesses the characteristics of non-linearity and non-uniformity. General signal processing methods, such as the Fourier transform, have difficulty dealing with this problem. Therefore, this paper introduces EEMD which is self-adaptive into the AKF to estimate the observation noise.

### 2.1. Ensemble Empirical Mode Decomposition

Huang invented a signal processing algorithm called EMD that can decompose a nonlinear signal into a series of near-orthogonal intrinsic mode functions (IMFs) [23]. EMD is an adaptive time–frequency data processing algorithm, which is widely used to extract signals of interest from non-linear and non-stationary processes containing noise [24,25]. However, it is well known that EMD suffers from a mode mixing problem, which is defined as signals of different frequencies contained in a single IMF, that is, the decomposition of the original signal is not sufficient [26]. To solve this problem, EEMD based on noise assistance is proposed [20]. 

The principle of EEMD is very complete.

Adding white noise of limited amplitude to the original data will help the work of EMD. Signals of different scales can be automatically projected onto appropriate IMF components [20]. This phenomenon depends on the statistical characteristics of the white noise itself [27].

Since the added white noise in each experiment is random, after averaging the results of enough experiments, the influence of the added white noise on the original signal will be eliminated [20]. 

By combining the original signal with white noise of limited amplitude, we can obtain a clearer IMF component [26]. The pseudo-code implementation of the EEMD is listed in Algorithm 1.

**Algorithm 1** EEMD**Input:** o(t): The original signal;
**begin**
 (1) $o^{\prime }(t)=o(t)+n(t)$//Add Gaussian noise series to the original signal o(t); n(t) is the Gaussian noise series with amplitude of $\sigma_{n}$; (2) $IMFs^{\prime }(t)=EMD(o^{\prime }(t))$//Decompose noise-added signal $o^{\prime }(t)$ into $IMFs^{\prime }(t)$ using EMD; (3) Repeat (1) and (2) M times with different Gaussian noise series which have the same amplitude; (4) Get the means of corresponding $IMFs^{\prime }(t)$ of the decompositions as the final IMFs(t);
**end**


As a result, the original signal is decomposed into the following form:

(1) $$o(t)=\sum_{f=1}^{F}IMF_{f}(t)+r(t)$$

where o(t) is the original signal, $IMF_{f}(t)$ is the *f*th IMF signal and r(t) is the residual signal.

### 2.2. Acquisition of Observation Noise Covariance Matrix R

The general steps of the acquisition of R are as follows:

The EEMD algorithm decomposes the GNSS signal within a fixed window size w into a series of IMF components (the introduction of the window makes the calculation of R pay more attention to the current noise situation instead of obtaining R from the data of the entire historical period, which wastes a lot of resources and is inaccurate). Then, the noise components are distinguished by comparing the power spectral density (PSD) of each component [21] and the original noise is reconstructed by the summation of the noise components. At last, the observed noise covariance matrix R is estimated by the standard deviation of the reconstructed noise.

Since EEMD requires a suitable window size w for the noise extraction, the setting of R is determined by the empirical value in the first w seconds, but the inaccuracy of R within a short period of time will not have a significant impact on the final result.

The calculation process of the observation noise covariance matrix R using EEMD is listed in Algorithm 2.

**Algorithm 2**$f_{EEMD}()$: observation noise covariance matrix by EEMD**Input:**
 $Z_{GNSS}$: a numeric sequence of GNSS signal;
 w: a window size used for computing *R*;**Output:**
 $R_{k}$: observation noise covariance matrix at time *k*;
**begin**
 (1) $IMFs:=EEMD(Z_{GNSS}[k-w:k])$//Decompose input signal using EEMD; (2) $IMF_{niose}:=Select(IMFs)$// Use power spectral density (PSD) to select IMF [21]; (3) $Z_{niose}:=Sum(IMF_{niose})$// Reconstruct the noise signal by summing $IMF_{niose}$; (4) $R_{k}:=CalculateVAR(Z_{niose})$// Calculate the variance of $Z_{niose}$; (5) Return $R_{k}$// return the estimated value of *R* at time *k*;
**end**


The inputs of this function are the GNSS signal $Z_{GNSS}$ and the window size w for filtering, and the output is *R* at time *k* which changes with the actual noise. 

### 2.3. INS Error Model

In this section, we will describe the INS error model in the east–north–up navigation reference frame. The error equation is as follows:

(1) Attitude error equation:
  (2) $${\dot{\varphi }}^{n}=\delta \omega_{ie}{}^{n}+\delta \omega_{en}{}^{n}+(\omega_{ie}{}^{n}+\omega_{en}{}^{n})\times \varphi^{n}-\delta \omega_{ib}{}^{n}$$
(2) Velocity error equation:
  (3) $$\delta {\dot{v}}^{n}=\varphi^{n}\times f^{n}-(2\delta \omega_{ie}{}^{n}+\delta \omega_{en}{}^{n})\times v^{n}-(2\omega_{ie}{}^{n}+\omega_{en}{}^{n})\times \delta v^{n}+\delta f^{n}$$
(3) Position error equation:
  (4) $$\begin{array}{l} \delta \dot{L}=-\frac{\delta v_{N}}{R_{M}+h}\\ \delta \dot{\lambda }=-\frac{\delta v_{E}}{R_{N}+h}\sec L+\frac{v_{E}}{R_{N}+h}\sec L\tan L\delta L\\ \delta \dot{h}=\delta v_{U} \end{array}$$

Navigation information errors include: the attitude error shown in the navigation frame $\varphi^{n}=(\varphi_{E}\;\varphi_{N}\;\varphi_{U})$ the velocity error $\delta v^{n}=(\delta v_{E}\;\delta v_{N}\;\delta v_{U})$ and the position error δP=(δL δλ δh). $f^{n}$ is the output of the accelerometer. $R_{M}$ and $R_{N}$ are the radii of the earth in the latitude and longitude directions, respectively. $\omega_{ie}{}^{n}$ and $\omega_{en}{}^{n}$ are the rotational angular velocities of the earth and navigation frame, respectively. $\delta \omega_{ib}{}^{n}$ and $\delta f^{n}$ are the errors of the gyro and accelerometer, respectively. $\delta \omega_{ib}{}^{n}$ consists of the gyro bias $\varepsilon^{n}$ and white noise $\omega_{g}{}^{n}$. $\delta f^{n}$ is composed of the accelerometer bias $\nabla^{n}$ and white noise $\omega_{a}{}^{n}$.

### 2.4. The Filtering Algorithm of Dynamic Adaptive Kalman Filter

Consider the following multivariable linear discrete system [28]:

(5) $$\begin{array}{l} X_{k}^{}=\Phi_{k/k-1}X_{k-1}^{}+W_{k-1}\\ Z_{k}=H_{k}X_{k}^{}+V_{k} \end{array}$$

where $X_{k}^{}$ is the state vector ($X_{k}^{}=[\delta P\;\delta v^{n}\;\varphi^{n}\;\varepsilon^{n}\;\nabla^{n}]$), $\Phi_{k/k-1}$ is the transition matrix formed by the error equation described in Section 2.3, $Z_{k}$ is the observation vector formed by the position difference of the GNSS receiver and the INS and $H_{k}$ is the observation matrix. $W_{k-1}$ and $V_{k}$ are the Gaussian white noise sequences satisfying the following conditions:

(6) $$\left\{ \begin{array}{l} E[W_{k}]=0,\;E[W_{k}W_{j}^{T}]=Q_{k}\begin{array}{cc} & j=k \end{array}\\ E[V_{k}]=0,\;E[V_{k}V_{j}^{T}]=R_{k}j=k\\ E[W_{k}V_{j}^{T}]=0 \end{array}\right.$$

where $Q_{k}$ is the process noise covariance matrix and $R_{k}$ is the observation noise covariance matrix.

The prediction equations are:

(7) $$\begin{array}{l} {\hat{X}}_{k/k-1}^{}=\Phi_{k/k-1}{\hat{X}}_{k-1}^{}\\ {\hat{P}}_{k/k-1}^{}=\Phi_{k/k-1}{\hat{P}}_{k-1}^{}\Phi_{k/k-1}^{T}+Q_{k} \end{array}$$

where ${\hat{X}}_{k/k-1}^{}$ is the predicted state vector; ${\hat{X}}_{k-1}^{}$ is the estimated state vector; ${\hat{P}}_{k/k-1}^{}$ is the predicted state covariance matrix; ${\hat{P}}_{k-1}^{}$ is the estimated state covariance matrix.

The update equations are:

(8) $$\begin{array}{l} K_{k}={\hat{P}}_{k/k-1}H_{k}^{T}{\left(H_{k}{\hat{P}}_{k/k-1}H_{k}^{T}+R_{k}\right)}^{-1}\\ {\hat{X}}_{k}={\hat{X}}_{k/k-1}+K_{k}^{}(Z_{k}-H_{k}{\hat{X}}_{k/k-1})\\ {\hat{P}}_{k}=(I-K_{k}H_{k}){\hat{P}}_{k/k-1}{\left(I-K_{k}H_{k}\right)}^{T}+K_{k}^{}R_{k}^{}K_{k}^{T} \end{array}$$

where $K_{k}^{}$ is the Kalman gain and $H_{k}$ is the observation matrix. It is worth mentioning that in order to ensure the positive semi-definiteness of ${\hat{P}}_{k}^{}$, we used its Joseph’s form [28].

In order to settle the uncertainty of the process noise, Ding et al. proposed an improving AKF based on the covariance matching method under the assumption that the observation noise is completely known and rewritten (7) as follows [10].

(9) $$\begin{array}{l} {\hat{X}}_{k/k-1}^{}=\Phi_{k/k-1}{\hat{X}}_{k-1}^{}\\ {\hat{P}}_{k/k-1}^{}=\Phi_{k/k-1}{\hat{P}}_{k-1}^{}\Phi_{k/k-1}^{T}+\sqrt{s_{k}}Q_{k-1} \end{array}$$

where $s_{k}$ is the scaling factor expressed as follows:

(10) $$s_{k}=\frac{trace\left\{\frac{1}{m}\sum_{e=0}^{m-1}d_{k-e}d_{k-e}^{T}-R_{k}\right\}}{trace\left\{H_{k}(\Phi_{k/k-1}P_{k-1}\Phi_{k/k-1}^{T}+Q_{k-1})H_{k}^{T}\right\}}$$

The innovation sequence $d_{k}$ is defined as:

(11) $$d_{k}=Z_{k}-H_{k}{\hat{X}}_{k/k-1}^{}$$

When $R_{k}$ is unknown or inaccurate, the calculation of the scaling factor will be affected. The adjustment of *Q* will become worse. When $R_{k}$ is accurately calculated by the proposed method, the solution of the scaling factor will be accurate. Therefore, *Q* will be adjusted more appropriately.

**Figure 1:**
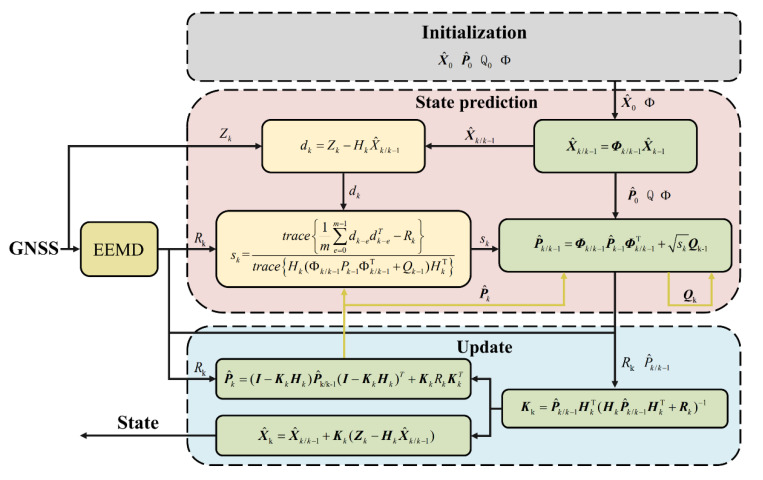
Process of Dynamic Adaptive Kalman Filter.

After substituting the $R_{k}$ calculated by Algorithm 2 into (10) used to solve the scaling factor, the complete process of DAKF is shown in Figure 1.

## 3. Network Navigation Algorithm

When the UAV swarm cannot receive the GNSS signal, the three-dimensional network navigation technology based on the distance information among all UAVs is introduced to reduce the error of the INS [19].

The specific process of the algorithm is as follows:

By linearizing the difference between the inertial distances calculated from the INS output and the measured distance between nodes, the following Equation can be obtained [29]:

(12) $$\begin{array}{l} l_{ij}=d_{ij}^{m}-d_{ij}^{I}\\ \approx -\frac{x_{i}^{I}-x_{j}^{I}}{d_{ij}^{I}}\Delta x_{i}^{I}-\frac{y_{i}^{I}-y_{j}^{I}}{d_{ij}^{I}}\Delta y_{i}^{I}-\frac{z_{i}^{I}-z_{j}^{I}}{d_{ij}^{I}}\Delta z_{i}^{I}\\ +\frac{x_{i}^{I}-x_{j}^{I}}{d_{ij}^{I}}\Delta x_{j}^{I}+\frac{y_{i}^{I}-y_{j}^{I}}{d_{ij}^{I}}\Delta y_{j}^{I}+\frac{z_{i}^{I}-z_{j}^{I}}{d_{ij}^{I}}\Delta z_{j}^{I}+\Delta_{ij} \end{array}$$

where $d_{ij}^{m}$ is the measured distance between nodes *i* and *j*, $d_{ij}^{I}$ is the inertial distance between nodes *i* and *j*, $l_{ij}$ is the difference between these two distances, $\left\{x_{i}^{I},y_{i}^{I},z_{i}^{I}\right\}$ is the INS output position of node *i* and $\left\{\Delta x_{i}^{I},\Delta y_{i}^{I},\Delta z_{i}^{I}\right\}$ is the position error of node *i*; it is assumed that the error of distance difference $l_{ij}$ obeys a normal distribution. This assumption is based on the fact that the noise of the INS and the error of the measured distance between nodes obey a normal distribution. $\Delta_{ij}$ is the error of distance difference $l_{ij}$, $\Delta_{ij}\sim N\left(0,\;\sigma_{r}{}^{2}\right)$ and $\sigma_{r}{}^{2}$ is the variance of the normal distribution [29].

The matrix form of (12) is [30]:

(13) $$\left\{ \begin{array}{l} L_{d}=TS+\Delta \\ E(L_{d})=0\\ \sum {}_{L_{d}}=\sigma_{0}^{2}P_{co}{}^{-1} \end{array}\right.$$

where $L_{d}$ is the p-dimensional measurement vector, T is the p × 3a dimensional coefficient matrix (a is the number of nodes), S is the 3a-dimensional position error vector, Δ is the p-dimensional measurement error vector, $\sigma_{0}^{2}$ is the unit weight variance and $P_{co}$ is the p × p dimensional measurement error covariance matrix of $L_{d}$.

According to the principle of least squares, Δ and S are replaced with their respective estimated values $V_{n}$ and $\hat{S}$. The model of the network navigation algorithm is established as follows [29]:

(14) $$\{\begin{array}{l} V_{n}=T\hat{S}-L_{d}\\ V_{n}{}^{T}P_{co}V_{n}=\min \end{array}$$

The above formula can be rewritten as follows:

(15) $$\hat{S}={\left(T^{T}P_{co}T\right)}^{-1}T^{T}P_{co}L_{d}$$

where $\hat{S}$ is the estimated position error vector.

However, the above formula can only prove that the shapes of the measurement network with measured distances between nodes as edges and the inertial position network with inertial distances calculated from the INS output as edges are the same.

To make the absolute positions of the two networks consistent in three-dimensional space, it is necessary to rotate and translate the two networks through the principle of the free network theory with rank deficiency mentioned in [29].

According to this principle, the above rotation and translation process is equivalent to adding the following new solution conditions [29]:

(16) $$G^{T}P_{X}\hat{S}=0$$

where $P_{X}$ is a 3a × 3a dimensional weight matrix determined by the accuracy level of each node’s inertial navigation system. The expression of $P_{X}$ is as follows:

(17) $$P_{X}=\left[\begin{array}{ccccccc} p_{x_{1}} & 0 & 0 & \cdots & 0 & 0 & 0\\ 0 & p_{y_{1}} & 0 & \cdots & 0 & 0 & 0\\ 0 & 0 & p_{z_{1}} & \cdots & 0 & 0 & 0\\ \vdots & \vdots & \vdots & \vdots & \vdots & \vdots & \vdots \\ 0 & 0 & 0 & \cdots & p_{x_{n}} & 0 & 0\\ 0 & 0 & 0 & \cdots & 0 & p_{y_{n}} & 0\\ 0 & 0 & 0 & \cdots & 0 & 0 & p_{z_{n}} \end{array}\right]$$

where $G^{T}$ is a matrix composed of six linearly independent eigenvectors corresponding to the zero eigenvalue of $T^{T}P_{co}T$. $G^{T}$ satisfies the following conditions:

(18) $$\{\begin{array}{l} \begin{array}{l} TG=0\\ T^{T}P_{co}TG=0 \end{array}\\ \mathrm{rank}\left(G^{T}\right)=6 \end{array}$$

The first item in (18) can ensure that the added solution conditions (16) and the error Equation (15) are independent of each other. It can be seen from the second item in (18) that G is composed of the zero eigenvectors of $T^{T}P_{co}T$. The third item in (18) means that G supplements the error Equation (15) with six constraints caused by rotation and translation [29].

The final estimated error of the node’s INS output can be expressed as:

(19) $$\hat{S}={\left(T^{T}P_{co}T+P_{X}GG^{T}P_{X}\right)}^{-1}T^{T}P_{co}L_{d}$$

According to the position S output by the INS and the error $\hat{S}$ estimated by the network navigation algorithm, the corrected value $\tilde{S}$ of the node position can be obtained:

(20) $$\tilde{S}=S-\hat{S}$$

The theory of the network navigation algorithm has been well established, and the specific details can be found in [30].

## 4. Simulation Evaluation

### 4.1. Simulation Configuration

The simulation uses four UAVs carrying the GNSS and INS, and employs the data link method to obtain the distances among all UAVs [31]. 

**Figure 2:**
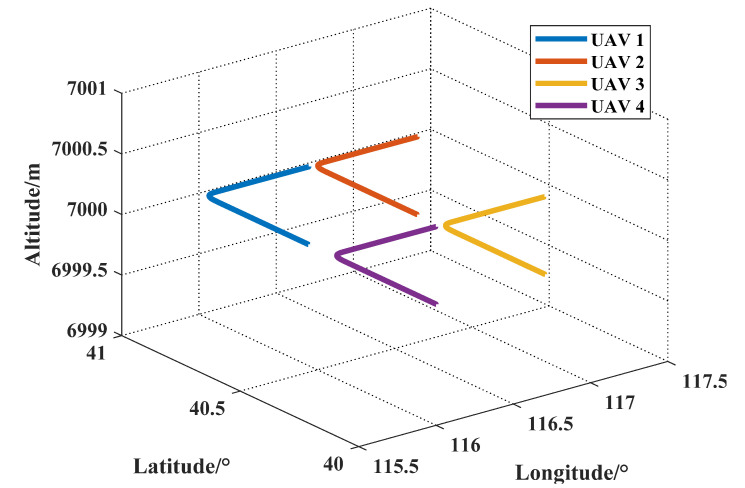
True trajectory of the UAVs.

At the beginning, four UAVs formed a square swarm with sides of 60 km. Subsequently, the swarm moved north and then east at a speed of 200 m/s. The true trajectory of the UAV swarm is shown in Figure 2. 

**Figure 3:**
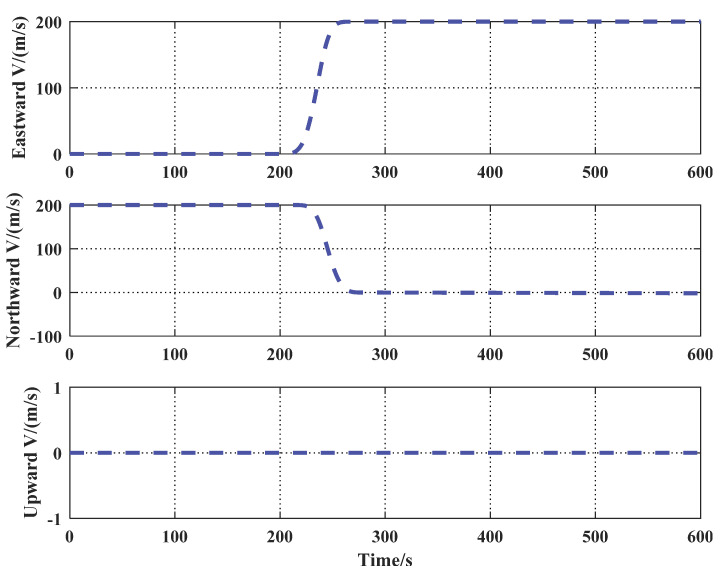
Velocity of UAV.

The velocity of the four UAVs in the east–north–up navigation reference frame is shown in Figure 3. (the change at about 220 s was caused by the movement of the UAV).

The initial attitude error was $\left(0.5^{\prime }\;0.5^{\prime }\;1.5^{\prime }\right)$, the initial velocity error was (0.5 m/s 0.5 m/s 0.5 m/s) and initial position error was (20 m 20 m 20 m). The bias and standard deviation of the white noise of the accelerometer were 800 μg and $100\;\mu g\cdot \sqrt{s}$. The bias and standard deviation of white noise of the gyro were 3°/h and $1{}^{\circ}/\sqrt{h}$. The standard deviation of the measurement error vector was 20 m. The process noise covariance matrix *Q* was set as:

(21) $$Q=diag[0_{3\times 3},{\left(100\;\mu g\cdot \sqrt{s}\right)}^{2}\cdot I_{3\times 3},{\left(1^{\circ }/\sqrt{h}\right)}^{2}\cdot I_{3\times 3},0_{6\times 6}]$$

### 4.2. Evaluation of Dynamic Adaptive Kalman Filter

In order to highlight the superiority of the proposed algorithm, two simulations were conducted. 

In the first simulation, the noise of the GNSS signal was designed as a step from 10 m to 30 m with the purpose of verifying the adaptability of the proposed algorithm. The parameters of EEMD were set as follows: M=20 and $\sigma_{n}=0.2$. 

**Figure 4:**
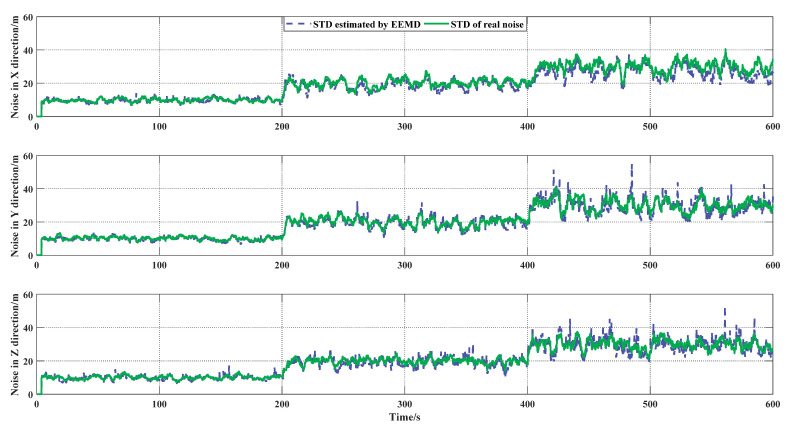
Proof of algorithm following effect. The green curve represents standard deviation (STD) of real noise, the blue curve represents STD estimated by EEMD.

It can be seen from Figure 4 that the proposed method could follow the changes of noise in three direction channels well. It is worth mentioning that in order to verify the effect of the proposed algorithm on noise reconstruction, this paper compares the standard deviation of the reconstructed noise and the real noise in the same window. Although the standard deviation of the real noise was set to three constant values, the real noise was actually a random value fluctuating around this constant value, due to the randomness of the noise itself [32]. Therefore, the green line is actually the standard deviation of the real noise calculated by the window at different positions. In addition, any digital signal processing method requires a window size for the accurate extraction of noise; therefore, errors in the first four seconds are allowed (w=4 s). 

The average difference between the two curves in Figure 4 is (X: 1.450 m Y: 0.574 m Z: 0.568 m)

The second simulation compared the classic Kalman filter with the proposed algorithm to prove the effectiveness of the DAKF. This simulation used the trajectory of UAV1 in Figure 2, and this method was also verified on the other three trajectories. 

Experimental parameters are shown in Table 1. In the first four seconds, the observation noise matrix R of DAKF was consistent with the KF, due to the introduction of the window in the proposed algorithm; in the following time, R of DAKF was dynamically obtained by EEMD and R of the KF was still a constant empirical value. In order to verify the universality of the proposed algorithm, setting the real noise of the three channels of GNSS included three stages: the real noise less than the model setting, the real noise greater than the model setting, and the real noise equal to the model setting. To ensure the effectiveness of the simulation, in Table 1, the real noise of the DAKF and KF were consistent. In other words, the data in the second row belong to the second and third columns together in Table 1.

**Figure 5:**
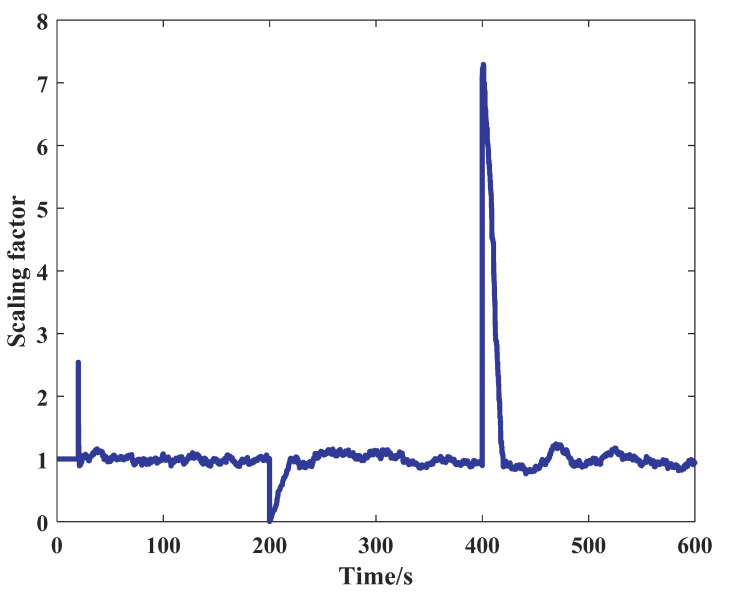
The change of scaling factor.

Figure 5 shows the change of the scaling factor. The fluctuation of the scaling factor at the initial moment is a normal phenomenon caused by the initial parameter setting which has been proven [10]. At 200 s and 400 s, it was used to prove the normal operation of the adaptive part that the scaling factor following the change of the GNSS noise [33]. Except for the above three transitional periods, the scaling factor was well maintained around one.

**Figure 6:**
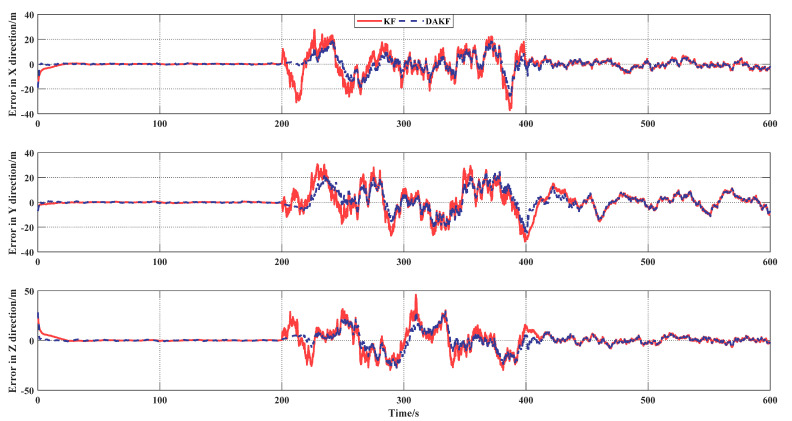
Position error of the two algorithms.

Figure 6 shows the simulation results of the KF and DAKF with dynamic observation noise. When the model noise did not match the real noise, the DAKF performed significantly better than the KF. When equal, the effects of the DAKF and KF were similar. A further explanation of Figure 6 is given by Table 2. Table 2 shows the average position error of the three channels, which proves the effective improvement of the DAKF.

### 4.3. Evaluation of Networked Navigation Algorithm

In order to fully verify the performance of the NNA, the UAV movement during the GNSS interruption should include two modes: straight and turning. The total time of the UAV swarm flying was designed to be 520 s while there was a GNSS outage of 150 s start at the 100th second. 

**Figure 7:**
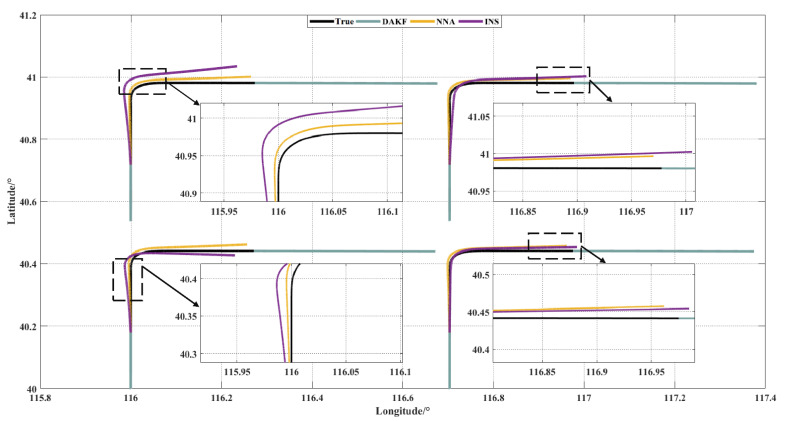
Swarm trajectory in the horizontal direction.

Since the swarm had no displacement in the vertical direction, Figure 7 shows the swarm trajectory in the horizontal direction in order to better show the effect of the NNA. The purple line is the navigation results of the INS. The yellow line is the trajectory after the position compensation of the NNA. The gray line is the output of the UAV’s INS/GNSS integrated system, which was basically consistent with the true trajectory, when the GNSS was available. The black line is the true trajectory shown in Figure 2. It can be easily obtained from Figure 7 that the NNA could obviously improve the navigation accuracy of the overall UAV swarm, no matter whether in the straight or turning mode.

Table 3 describes the improvement in positioning accuracy of the UAV swarm after using the NNA. Δp represents the position error of four UAV’s inertial navigation system and $\Delta \tilde{p}$ represents the position error after the compensation of the NNA. In this simulation, the above position error was calculated from the last moment of the GNSS signal being unavailable. The expression is as follows:

(22) $$\begin{array}{l} \Delta p_{i}=\sqrt{{(\Delta x_{i}^{I})}^{2}+{\left(\Delta y_{i}^{I}\right)}^{2}+(\Delta z_{i}^{I}{)}^{2}}\\ \Delta {\tilde{p}}_{i}=\sqrt{{(\Delta {\tilde{x}}_{i}^{I})}^{2}+{\left(\Delta {\tilde{y}}_{i}^{I}\right)}^{2}+(\Delta {\tilde{z}}_{i}^{I}{)}^{2}} \end{array}i=1,2,3,4$$

where $\Delta {\tilde{x}}_{i}^{I},\Delta {\tilde{y}}_{i}^{I}$ and $\Delta {\tilde{z}}_{i}^{I}$ are the position errors in three directions after the NNA compensation of the ith aircraft.

To define the improvement of positioning accuracy as the ratio of Δp and $\Delta \tilde{p}$:

(23) $$P_{imp}=\frac{\Delta p}{\Delta \tilde{p}}$$

Table 3 shows that the NNA could increase the positioning accuracy of the UAV swarm by 1.93 times. In addition, the purpose of the NNA was to improve the positioning accuracy of the entire swarm. The deterioration of the positioning accuracy of a single node (UAV3) due to the change of the swarm’s shape which was caused by the divergence of the INS navigation errors with time was allowed [19,34].

In order to verify the superiority of the proposed algorithm compared with the neural network-based algorithm, the NNA and MLP-based Bagging method mentioned in [18] were compared on UAV1 (the simulation result was similar on the other three UAVs). The parameter settings and experimental results of the NNA were consistent with the above simulation. The topology and transfer function of the MLP were 12 (input layer) × 6 (hidden layer) × 1 (output layer) and logsig-linear, respectively. The network number of Bagging was set to 50. The training data of the neural network were UAV information during the first 100 s when the GNSS signal was available. The output of the MLP was the increment of the UAV’s position during the GNSS outage.

**Figure 8:**
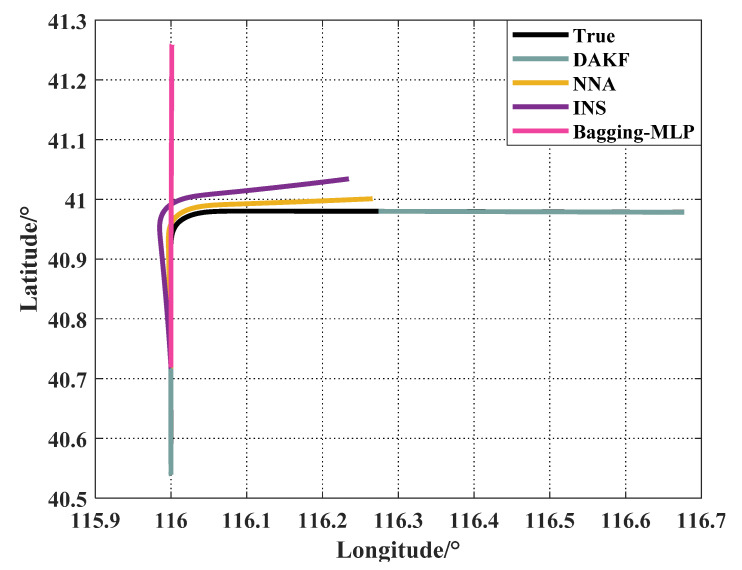
Comparison results between NNA and MLP-based Bagging method.

Figure 8 shows the comparison result between the NNA and MLP-based Bagging method. The pink line is the prediction result of the MLP-based Bagging method.

The positioning error of the MLP-based Bagging was 6385.014 km, which was approximately 41 times larger than the positioning error of the NNA. One of the disadvantages of the neural network-based algorithm is that it relies heavily on training data. If the training sample and the test sample were significantly different, the above situation would be an inevitable phenomenon [18]. In contrast, the NNA was only related to the distance between nodes at the current moment, so it would not be affected by historical data. It can be seen from the simulation results that the NNA was more robust than the neural network-based methods.

## 5. Conclusions

This paper proposed a new navigation scheme for the UAV swarm. The proposed algorithm included the following two cases: (1) By adaptively acquiring the observation noise covariance matrix using EEMD and adjusting the process noise covariance matrix, a dynamic adaptive Kalman filter which is superior to the traditional Kalman filter was designed when the GNSS signal was available. The simulation shows that the DAKF could effectively improve the accuracy of the Kalman filter. (2) In the phase of GNSS signal outage, the positioning error of the UAV swarm was reduced through the use of the networked navigation algorithm. The simulation demonstrated that the NNA could improve the positioning accuracy of the swarm by 93% during the GNSS interruption of 150 s and better than the neural network-based algorithm. 

Future work mainly includes the following research directions. First, the colored noise and non-Gaussian noise were not considered in this simulation. It would determine whether the proposed algorithm has a sufficient generality in all environments. Second, the window size of the DAKF was an empirical value obtained from a large number of experiments. How to decrease its impact on the algorithm will be considered in the next step. Finally, the proposed algorithm has not been verified on an actual UAV swarm, which will be carried out in future work.

## Figures and Tables

**Table 1:** Experimental parameters.

	DAKF	KF
$R_{model}/m$	Dynamic	(30,30,30)
Real noise/m	(2,2,2)→(100,100,100)→(30,30,30)	

**Table 2:** Average position error.

	DAKF	KF	Improvement
X/m	24.1702	52.4235	54%
Y/m	48.8251	74.2594	34%
Z/m	49.2883	74.1551	34%

**Table 3:** Improvement in positioning accuracy of NNA.

	$\Delta \tilde{p}/km$	Δp/km	$P_{imp}$
UAV 1	157.142	460.443	2.93
UAV 2	102.436	224.773	2.20
UAV 3	127.555	96.923	0.76
UAV 4	177.985	307.256	1.73
Average	141.280	272.349	1.93

## Data Availability

Not applicable.
